# Lithium-Induced Awakening of Neural Cell Memory of Involuntary Dyskinesia: A Case Report

**DOI:** 10.7759/cureus.42592

**Published:** 2023-07-28

**Authors:** Rajnish Raj, Manpreet Kaur Virk

**Affiliations:** 1 Department of Psychiatry, Government Medical College and Rajindra Hospital, Patiala, IND

**Keywords:** naranjo adverse drug reaction probability scale, lithium, involuntary dyskinesia, dopamine, cell memory

## Abstract

Involuntary movement disorders include tremors, tics, myoclonus, athetosis, chorea, dystonia, and dyskinesia. Neuroleptic drugs have the propensity to cause extrapyramidal side effects. Lithium-induced coarse tremors are well documented and may occur at therapeutic serum concentrations (0.8-1.0 mEq/L) in the treatment of bipolar disorder. Treatment for coarse tremors due to lithium includes either dose reduction or non-selective beta-blockers. To our knowledge, there are only four case reports regarding the lithium-induced awakening of cell memory of involuntary movement disorders worldwide. In scientific literature, only two drugs have the propensity to reawaken past cell memory. These intriguing findings can have a wider application across fields such as past-life regression therapy, post-traumatic stress disorder, catharsis, or recall of sub-aural temporal high-frequency burst-erased memory-type of mind-altering techniques. We report a case of lithium-induced awakening of the cell memory of involuntary dyskinesia in a female who took treatment for bipolar disorder in the past.

## Introduction

There are various movement disorders, namely, dystonia, tremor, myoclonus, tics, hemiballismus, chorea, athetosis, and tardive dyskinesia (TD). All of these have multiple etiological factors, for example, genetics, toxins, metabolic abnormalities, infections, and stroke [1]. Among them, neuroleptic drugs are one of the most important causes. Neurons of forgotten pasts and hidden patterns can be reactivated by re-exposure to drugs used before, discontinuation of drugs to which neurons have been chronically exposed, or sometimes by the introduction of new agents that unveil the previously altered cellular mechanisms [2]. Lithium carbonate is the first-line drug for bipolar disorder type I and for manic-depressive illness cyclic pattern. It is used for both the treatment and prophylaxis of manic or depressive states in bipolar affective disorder [3]. The notable side effects are nausea, vomiting, diarrhea, weight gain, tremors, ataxia, dysarthria, polyuria, polydipsia, acne, systematic lupus erythematosus, leucocytosis, and hypothyroidism [4]. A few cases of lithium-induced reawakening of past cell memory of involuntary movement disorder have been reported in the literature [5]. Here, we report a rare case of an already-treated female who developed lithium-induced involuntary dyskinesia.

## Case presentation

A 32-year-old female reported to the psychiatry outpatient department (OPD) with chief complaints of decreased need for sleep, overactivity, overspending, and grandiosity for the last two weeks. She was diagnosed case of bipolar affective disorder for the last six years. Per her past records, she had two similar episodes. The first episode of mania was sudden in onset. She had presented with grandiosity, over-talkativeness, overactivity, over-familiarity, spending spree, and decreased need for sleep six years ago when she was 27 years old. At that time, she was treated with an oral dose of tablet divalproex sodium 500 mg twice daily, tablet haloperidol 5 mg twice daily, and tablet lorazepam 2 mg at night. Haloperidol was gradually stopped, but a maintenance dose of divalproex sodium 500 mg twice daily was continued for six months with the occasional use of oral tablet benzodiazepine for sleep. The second episode of mania occurred after one year. At that time, an oral dose of tablet lithium 900 mg per day, a tablet divalproex sodium 500 mg twice daily, a tablet haloperidol 5 mg at night, and a tablet risperidone 3 mg plus trihexyphenidyl 2 mg twice daily were given. Haloperidol and risperidone plus trihexyphenidyl were gradually tapered down and stopped over a period of four weeks, and thereafter, maintained well on divalproex sodium 500 mg twice daily and benzodiazepine treatment. For the current episode, on general physical examination, she had hirsutism. Per-abdomen ultrasound examination revealed no evidence of polycystic ovarian disease. Mental status examination showed the patient was conscious and well-oriented to time, place, and person. She was dressed in gaudy and bright clothes according to the weather, and her speech was increased in rate, rhythm, and volume. The mood was elated, and affect was appropriate to the mood content, full in range and intensity. The thought process had a flight of ideas; the content revealed delusion of the grandiosity of self-worth, power, and self-referential paranoid delusion. The higher mental functions for attention, concentration, memory, and calculations were intact. However, judgment and abstract thinking were impaired, and insight into the illness was absent.

According to the International Classification of Diseases 10th Edition, she was diagnosed as a case of bipolar affective disorder, with a current episode of mania with psychotic symptoms (F31.2). She was given a conventional antipsychotic therapy consisting of an oral dose of haloperidol 5 mg at night and a tablet of lithium 300 mg twice daily and a tablet of lorazepam 2 mg at night. After two weeks of treatment, the patient manifested de novo involuntary movements in the form of tremors, shoulder shrugging of upper limbs, eye blinking, and twitching of the face that were precipitated by anxiety but abated during sleep. There was no history of fever, autonomic instability, or “marche à petit pas” movement of lower limbs. Various differential diagnoses of tics, choreoathetosis, dyskinesia, or neuroleptic malignant syndrome were kept in mind. Routine blood investigation including total leucocyte count, differential leucocyte count, complete blood count, electrolytes, serum lithium level of 0.4 mEq/l, and C-reactive protein kinase levels were within normal limits. Magnetic resonance imaging of the brain was also normal. The Abnormal Involuntary Movements Scale (AIMS) showed a score of 9, indicating mild evidence of TD. The causality assessment on Naranjo adverse drug reaction (ADR) probability scale showed a score of 7, indicating probable ADR. Haloperidol was stopped and an oral dose of tetrabenazine 25 mg in three divided doses was added, but the patient’s symptoms showed no improvement. Propranolol was given to attenuate anxiety, but to no avail. The patient complained of hair loss and hirsutism. Divalproex sodium was stopped gradually and a tablet biotin 10 mg once daily was added. However, lithium was continued. The symptoms of shoulder shrugging, eye-blinking, neck cracking, and throat clearing continued, and the Yale global tic scale score was 30. After one month of adequate trial and two weekly periodic follow-ups, the patient’s condition did not improve. Tablet clonidine 0.1 mg twice daily was added with low-dose tablet haloperidol 0.25 mg twice daily. Within 24 hours, her symptoms of involuntary movements were exaggerated, and her AIMS score was 16. It was certain that these symptoms were not due to tics. The possibility of dyskinesia was kept, and an injection of promethazine 25 mg/mL, 50 mg intramuscular stat was given. Some improvement was noted. Considering the response criteria of more than 50% reduction of the symptoms after therapeutic intervention, the results were unsatisfactory. Tablet propranolol 20 mg twice daily was given to control tremors, but it failed to give any respite to the patient. Tablet lithium 300 mg twice daily was the only offending drug left, which raised the suspicion of its possibility of perpetuating involuntary dyskinesia, and therefore, it was stopped. Tablet trihexyphenidyl 4 mg in divided doses, tablet oxcarbazepine 150 mg per day in two divided doses, and lorazepam 2 mg at night were given. After about seven days of treatment, involuntary dyskinesia significantly improved, and the Clinical Global Impression (CGI-I) scale score was 1 (significantly improved). However, manic symptoms re-emerged, possibly due to inadequate serum therapeutic levels of oxcarbazepine during cross-titration. NHS Maudsley clinical psychiatric practice guidelines were adhered to and lithium was re-instituted. However, within about five days of starting the treatment, involuntary dyskinesia re-emerged, and the CGI-I score was 6 (significant worsening) after therapeutic intervention with an efficacy index of 16, indicating side effects outweighing the benefits. Naranjo ADR scale score of 9 on the re-challenge test also established definite causality to lithium. Lithium was withdrawn and the patient was given tablet oxcarbazepine 450 mg per day in two divided doses, tablet trihexyphenidyl 4 mg in two divided doses, and tablet lorazepam 2 mg at night. On subsequent bi-weekly follow-ups for three months, she remained symptom-free of mania and dyskinesia. The patient rejoined her profession, delivered lectures, had public appearances, started earning, and reported satisfaction.

## Discussion

This is the fifth case report of lithium-induced involuntary movement disorder. Cases have been reported on the side effects of lithium on neostriatum leading to abnormal involuntary movements such as choreoathetosis, tardive dystonia, and parkinsonism [6,7]. The neo-striatal pathology could be the possible explanation regarding accidental exposure to a toxic build-up of intracellular concentrations of lithium (both intracellular and extracellular) and its substitution of sodium salts over time by altering membrane permeability and electric transmissibility, thus causing dyskinesia. In this case, lithium’s temporal association and dyskinesia re-appearance after the re-challenge test established definite causality. Such patients often have preexisting insults of basal ganglia due to decreased dopaminergic receptor density by pharmacological blockade or cell death that can trigger bipolar disorder or involuntary movement disorder. In addition, lithium has the propensity to rekindle past neuronal insult either due to streptococcal infection [2] or awaken quiescent cell memory of involuntary movement disorder by neuroleptics [5]. TD is a chronic and disabling disease, and its incidence in bipolar patients occurs in the range of 19% to 23% [8]. It exhibits neurotoxicity at low doses. Moreover, motor symptoms worsen during depression but disappear during mania. Furthermore, oro-lingual dyskinesia responds well to electroconvulsive therapy compared to limb-axial involvement of TD in bipolar disorder. Therefore, psychiatrists should be aware of such a rare phenomenon and stop the offending drug, herein lithium, vis-a-vis cross-titrate to other pharmacological agents for the management of bipolar affective disorder.

## Conclusions

Lithium-induced awakening of cell memory of involuntary dyskinesia may occur in female gender, younger individuals, and bipolar disorder type I patients, either with antecedent streptococcal infection or due to conventional antipsychotics that block dopamine receptors, but increase post-synaptic super-sensitivity of its receptors, thus causing dyskinesia. It may share bimodal diathesis with Parkinson’s disease leading to early onset of dementia and death by suicide. Therefore, early detection and prompt intervention are crucial in patient care.
